# C20209T prothrombin gene mutation associated deep venous thrombosis in a hemodialysis patient 

**DOI:** 10.5414/CNCS107984

**Published:** 2014-01-15

**Authors:** Samer Bani-Hani, Omar Siddiqui, Anami Patel, Arif Showkat

**Affiliations:** 1 University of South Florida, Tampa, FL,; 2 University of Tennessee Health Sciences Center (UTHSC), and; 3 Molecular Diagnostics Laboratory, LeBonheur Children’s Hospital, Memphis, TN, USA

**Keywords:** end-stage renal disease, deep vein thrombosis, C20209T prothrombin gene mutation

## Abstract

Venous thromboembolism (VTE) represents the formation of a blood clot in one of the deep veins of human body. The significant morbidity and mortality rates associated with VTE have spurred increasing investigations seeking to identify causative factors for this complex condition. While the most frequent causes of an inherited hypercoagulable state are the Factor V Leiden mutation and the prothrombin gene mutation, polymerase chain reaction (PCR) analysis has helped to identify other rare causes of inherited VTE. We report a case of a recurrent deep venous thrombosis in an end-stage renal disease patient. All laboratory tests for hypercoagulable states were normal. However, PCR analysis detected a rare polymorphism of prothrombin gene mutation at position C20209T, instead of G20210A. The patient was treated successfully with a high dose of warfarin to maintain adequate anti-coagulation during the 2-year follow-up.

## Introduction 

Venous thrombosis is the formation of thrombi in the deep veins. It affects 0.1% of persons per year and is a potentially dangerous condition with a myriad of risk factors [1]. The most common presentations of venous thrombosis are deep vein thrombosis (DVT) of the lower extremity and pulmonary embolism. The most frequent causes of an inherited (primary) hypercoagulable state are the Factor V Leiden mutation and the prothrombin gene mutation (G20210A), which together account for 50 – 60% of cases [2, 3]. 

We report a case of DVT in an end-stage renal disease (ESRD) patient due to rare polymorphisms of prothrombin gene mutation at position C20209T instead of G20210A. 

## Case history 

A 30-year-old African-American female with a past medical history of hypertension and ESRD presented to the emergency room complaining of new-onset neck swelling and pain. She denied any family history of DVT or loss of pregnancy. Her vital signs were: temperature 37 °C, blood pressure 152/85 mmHg, heart rate 78 beats/min, and respiratory rate 20 breaths/min. On examination, there was swelling on the left side of the neck with mild tenderness to palpation. She had a permanent dialysis catheter in the left internal jugular vein. Chest auscultation revealed normal S1 and S2 without any murmur, gallop, or rub. The lungs were clear to auscultation bilaterally and the abdomen was soft and non-tender. The patient did not have any peripheral edema or cyanosis. 

Laboratory results on admission showed: sodium 132 mEq/L, potassium 6.9 mEq/L chloride 89 mEq/L, carbon dioxide 27 mEq/L BUN 62 gm/dL, glucose 68 mg/dl, calcium 8.4 mg/dL, creatinine 11.4 mg/dL, total protein 8.6 g/dL, albumin 3.5 g/dL, and total bilirubin 1.1 mg/dL. White blood cell count was 14.3 × 10^3^/µL, hemoglobin 15.2 g/dL, hematocrit 45.2%, and platelet count 154 × 10^3^/µL. Neutrophil count was 80.8%, lymphocytes 8%, and monocytes 10.2%. 

Doppler ultrasound showed left internal jugular vein thrombosis. She was started on an intravenous heparin drip followed by oral warfarin treatment. Warfarin was discontinued after 3 months of treatment. Subsequently, the patient’s left arm brachiocephalic arteriovenous fistula became functional and the left internal jugular dialysis catheter was removed. Later on, the patient developed another episode of DVT involving the right jugular vein despite any history of venous instrumentations. Thrombophilia screening tests were sent including prothrombin time, activated partial thromboplastin time, thrombin time, fibrinogen, antithrombin activity, protein C activity, protein S activity, lupus anticoagulant, anticardiolipin antibodies, homocysteine, activated protein C resistance, Factor V Leiden, and the prothrombin G20210A gene mutation. All of the aforementioned tests were negative. However, polymerase chain reaction (PCR) and fluorescence resonance energy transfer detected one copy of another prothrombin gene mutation C20209T. The specimen preparation was performed by using the Qiagen EZ1 DNA Blood Kit and amplification/detection was done by using Roche Factor II (Prothrombin) G20210A Kit on LightCycler Instrument. A165 bp fragment of the Factor II gene was amplified from human genomic DNA using specific primers. The amplicon was detected by fluorescence using a specific pair of H probes consisting of two different oligonucleotides that hybridize to an internal sequence of the amplified fragment during the annealing phase of the PCR cycle. One probe was labeled at the 5ʼ-end with LightCycler Red 640-N-hydroxy-succinimide ester and, to avoid extension, modified at the 3ʼ-end by phosphorylation. The other probe was labeled at the 3ʼ-end with fluorescein. 

The patient resumed warfarin to maintain therapeutic international normalized ratio (INR) of 2.0 – 3.0. She remained free from any new episode of DVT during the 2 years of follow-up. Her average warfarin requirement was 12 – 14 mg per day. 

## Discussion 

The causes of venous thrombosis can be divided into two groups: hereditary and acquired. The major acquired risk factors for venous thromboembolism (VTE) include recent major surgery, trauma, immobilization, lupus anticoagulant and elevated levels of antiphospholipid antibodies, malignancy, pregnancy, oral contraceptives, and myeloproliferative disorders [5, 6, 7]. 

Inherited thrombophilia has a genetic tendency to VTE. The Factor V Leiden and prothrombin G20210A mutations are the most common defects, accounting for more than 50% of cases. Deficiencies in protein S, protein C, and antithrombin III account for most of the remaining cases, while rare causes include dysfibrinogenemias [8, 9]. 

Prothrombin (Factor II) is the precursor of thrombin, the end product of coagulation cascade. It is a vitamin K-dependent protein, which is synthesized in the liver and circulates with a half-life of ~ 3 – 5 days. Vitamin K acts as a cofactor for posttranslational γ-carboxylation of prothrombin, which is required for functional activity. 

Numerous gene mutations in various molecules have been found in members of families with inherited thrombophilia, but many mutations remain unidentified [10]. Prothrombin gene mutation G20210A is one of the hereditary risk factors associated with increased risk of VTE. It was first described by Poort et al. [11] to be a moderate risk factor for DVT and present in ~ 6% of patients with history of VTE. However, many other prothrombin gene mutations lead to bleeding tendencies, such as prothrombin deficiencies, dysprothrombinemia and hypoprothrombinemia. 

Prothrombin A19911G appears to raise prothrombin levels by increasing splicing efficiency at the nearby 20210 splice site [12]. Von Ahsen et al. [13] suggested that this polymorphism slightly increased the risk of VTE in carriers of the G20210A genotype, while Pérez-Ceballos et al. [14] concluded that it increased the risk of VTE in carriers of Factor V Leiden as well as in those without thrombophilia in their study. 

Miyawaki et al. [15] identified a novel mechanism of hereditary thrombosis associated with antithrombin resistance in a Japanese family, with a substitution of arginine for leucine at position 596 in the gene encoding prothrombin (called prothrombin Yukuhashi). It was postulated that this mutation results in slightly impaired yet adequate procoagulant function of the mutant prothrombin, but considerably impaired inhibition of the mutant thrombin by antithrombin. The antithrombin-resistant thrombin may have prolonged procoagulant activity in vivo, conferring a susceptibility to thrombosis. 

Initial reports of prothrombin gene mutation C20209T differ as to whether this polymorphism is [16, 17] or is not [18] associated with an increased risk for development of VTE. 

Warshawsky et al. [17] described the heterozygous detection of the base exchange C→i T 1 bp upstream, at position 20209 of F2, in three unrelated African-American individuals with a history of VTE events. Van der Putten et al. [18] concluded that the prothrombin C20209T mutation was probably not an important risk-modifier of VTE in African-Americans. However, in his pilot study Arya showed that C20209T variant is more common among African-Americans than it is among Caucasians [19]. Molecular studies have shown that this mutation is associated with gain-of-function of 3ʼ-end processing and up regulation of prothrombin protein expression as assessed by a highly sensitive luminescence-based reporter system [20]. 

Other risk factors for this variant include age less than 50 years and female gender. Serum prothrombin levels were elevated in these patients [21]. The exact incidence of the gene mutation is not well known [17, 22]. The C20209T prothrombin gene mutation can be detected by PCR methods. 

Anti-coagulation, which prevents further clot extension, acute pulmonary embolism, recurrence of thrombosis, and the development of late complications, such as post-thrombotic syndrome and chronic thromboembolic pulmonary hypertension, is the standard treatment for acute DVT. It is recommended that patients receive a parenteral anticoagulant (such as LMWH, fondaparinux, or unfractionated heparin) for at least 5 days and to start vitamin K antagonist (VKA) treatment the same day. The parenteral anticoagulant is recommended until the INR is ≥ 2.0 for 24 hours minimum; or, if the INR is > 3.0, the parental anticoagulant treatment can stop early [23]. The duration of VKA therapy is determined in part by the presence or absence of persistent risk factors and whether the DVT represents a first or a recurrent episode. 

Our patient had a recurrence of DVT after termination of initial long-term anti-coagulation therapy. Since resuming anti-coagulation, she has remained free from any recurrence. Her arteriovenous access for hemodialysis also remained functional without any episode of thrombosis. She did require a fairly high dose of warfarin to maintain therapeutic INR. This possibly reflects the effect of elevated prothrombin levels due to the genetic mutation. The testing and reporting of this rare variant of prothrombin gene mutation by the laboratory and the management practice is not well established. Atta et al. [24] suggested that prothrombin gene mutation should be tested in a patient with DVT in those who have recurrences of DVT, in patients with a positive family history or in those who demonstrate warfarin-resistance. Patients affected with this gene mutation should receive higher doses of warfarin (to reach the intended levels of INR) for a longer period of time. 

To our knowledge this is the first confirmed ESRD patient with recurrent DVT due to C20209T prothrombin gene mutation. 

## Conflict of interest 

The authors declare no financial conflicts.
